# MAPK, NFκB, and VEGF signaling pathways regulate breast cancer liver metastasis

**DOI:** 10.18632/oncotarget.20843

**Published:** 2017-09-12

**Authors:** Xinhua Chen, Zhihong Zheng, Limin Chen, Hongyu Zheng

**Affiliations:** ^1^ Department of Medical Oncology, Fujian Cancer Hospital & Fujian Medical University Cancer Hospital, Fuzhou, Fujian, China; ^2^ Department of Hematology, Fujian Medical University Union Hospital, Fuzhou, Fujian, China

**Keywords:** breast cancer, metastasis, liver, microarray, interaction network

## Abstract

In this study, we investigated the molecular pathways regulating breast cancer liver metastasis. We identified 48 differentially expressed genes (4 upregulated and 44 downregulated) by analyzing microarray dataset GSE62598 from Gene Expression Omnibus (GEO). We constructed a genetic interaction network with 84 nodes and 237 edges using the String consortium database. The network was reliably robust with a clustering coefficient (cc) of 0.598 and protein-protein interaction (PPI) enrichment *p* value of zero. Using the Gene Ontology and Kyoto Encyclopedia of Genes and Genomes databases, we identified MAPK, NFκB and VEGF signaling pathways as the most critical pathways regulating breast cancer liver metastasis. These results indicate that the distinct breast cancer metastatic stages, including dissemination from the primary breast tumor, transit through the vasculature, and survival and proliferation in the liver, are regulated by the MAPK, NFκB, and VEGF signaling pathways.

## INTRODUCTION

Breast cancer is the most frequently diagnosed cancer globally and is the leading cause of cancer-related deaths among women [1]. In the United States, more than 240,000 newly diagnosed breast cancer cases and 40,000 deaths were reported in 2016 [2]. Liver metastasis is reported in 15% of newly diagnosed breast cancer patients [3, 4]. Breast cancer liver metastasis is associated with very poor prognosis and has a survival time of only 4-8 months, if untreated [5]. Introduction of new therapies in the last decade has resulted in 1-2% yearly decrease in mortality rates [6]. However, breast cancer patients with liver metastasis still are associated with very poor outcomes [7].

Metastatic disease is a complex, multistage process that involves detachment of breast cancer cells from the primary tumor, which then travel through the blood or lymphatic system and finally survive and proliferate in the liver. Given the complex multistep process, liver metastasis involves a sophisticated network of molecular events. However, the molecular mechanisms associated with breast cancer metastasis to the liver are unclear, and their understanding is essential for developing more effective therapies. In this study, therefore, we generated a genetic interaction network using microarray gene expression data from breast cancer liver metastases and explored the molecular mechanisms involved using bioinformatic analyses.

## RESULTS

### Forty-eight genes are differentially expressed in metastatic breast tumor cells

**Figure 1:**
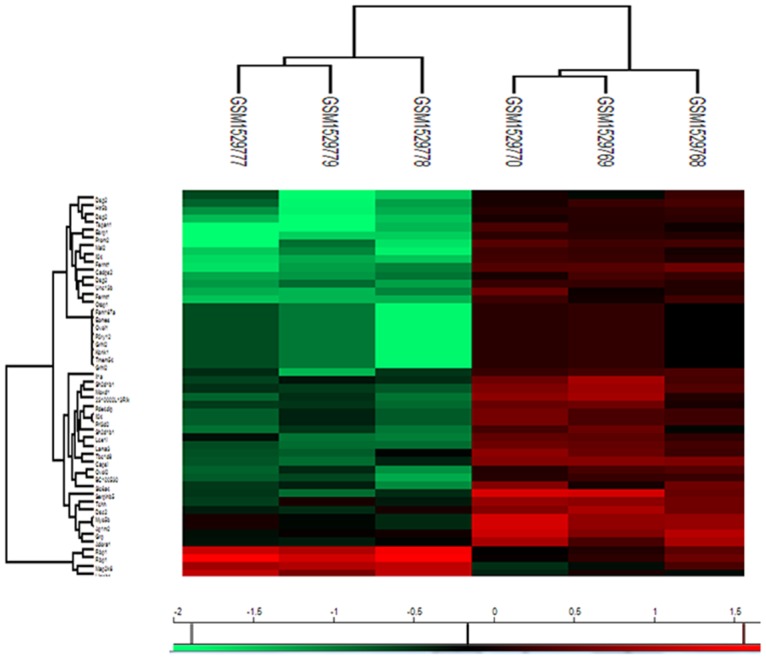
Heatmap visualization of the differently expressed genes identified by Significant Analysis of Microarray (SAM) in metastatic tumor cells (GSM1529777, GSM1529778, GSM1529779) versus 4T1 parental cells (GSM1529768, GSM1529769, GSM1529770) Red represents up-regulated genes, while green represents down-regulated genes.

Table 1 lists the differentially expressed genes with a fold change ≥2 and false discovery rate ≤ 5%. There were 48 differentially expressed genes that were distinctly upregulated (4 genes) or downregulated (44 genes) in metastatic tumor cells than in normal parental cells. Figure 1 shows the heat map of the differentially expressed genes.

**Table 1:** Significant genes identified by significant analysis of microarray (SAM) in liver-aggressive explant versus primary tumor explant

Gene ID	Gene Name	Fold Change	Gene regulation
A_52_P618173	*Limch1*	2.290749902	Up
A_52_P418791	*Rbp1*	2.424147188	Up
A_51_P423484	*Rbp1*	2.165856946	Up
A_52_P299915	*Map2k6*	2.176087369	Up
A_51_P102538	*Otop1*	0.336723951	Down
A_51_P289341	*Fermt1*	0.317362329	Down
A_52_P452667	*Prom2*	0.285970233	Down
A_51_P333923	*Tspan1*	0.315241505	Down
A_51_P167489	*Lama3*	0.41612039	Down
A_51_P177242	*Unc13b*	0.418318499	Down
A_52_P88091	*Dsg2*	0.403969687	Down
A_51_P233153	*Cadps2*	0.298078637	Down
A_51_P196207	*Capsl*	0.388252581	Down
A_52_P79821	*Esrp1*	0.26893644	Down
A_52_P559779	*Dsg2*	0.347328438	Down
A_51_P493987	*Moxd1*	0.417459194	Down
A_52_P87757	*Il24*	0.336785971	Down
A_52_P134455	*Fermt1*	0.367135842	Down
A_51_P356055	*Grp*	0.449573589	Down
A_51_P353252	*Mal2*	0.291415896	Down
A_51_P187602	*Serpinb5*	0.3120555	Down
A_52_P638605	*Ap1m2*	0.436913739	Down
A_51_P105879	*Myo5b*	0.486596961	Down
A_52_P405945	*Prl3d2*	0.483474132	Down
A_51_P401517	*Il24*	0.483144818	Down
A_52_P252931	*Dsc2*	0.491809463	Down
A_52_P468068	*Tchh*	0.490774711	Down
A_51_P322115	*Htr5b*	0.372641522	Down
A_52_P286350	*Sh2d1b1*	0.471867312	Down
A_52_P487686	*BC100530*	0.483518325	Down
A_51_P489488	*Pde4dip*	0.487698119	Down
A_51_P179293	*2310002L13Rik*	0.382311761	Down
A_51_P322090	*Ovol2*	0.489037358	Down
A_52_P661412	*Adora1*	0.485167002	Down
A_52_P683580	*Tbc1d9*	0.471654273	Down
A_51_P206475	*Lce1i*	0.476512201	Down
A_51_P496540	*Sh2d1b1*	0.488430246	Down
A_52_P601757	*Dsg2*	0.414988774	Down
A_51_P496253	*Slc6a4*	0.464974691	Down
A_51_P438283	*Il1a*	0.497937489	Down
A_51_P455620	*Fam167a*	0.45781262	Down
A_51_P332309	*Eomes*	0.434829918	Down
A_51_P225827	*Ovol1*	0.474676527	Down
A_51_P338878	*P2ry12*	0.424196491	Down
A_52_P373982	*Grhl2*	0.481346604	Down
A_52_P642488	*Kcnk1*	0.43461204	Down
A_51_P303079	*Tmem54*	0.492962995	Down
A_51_P362328	*Grhl2*	0.469572322	Down

**Abbreviation:** SAM, Significance Analysis Microarray

### A genetic interaction network based on the differently expressed genes

**Figure 2:**
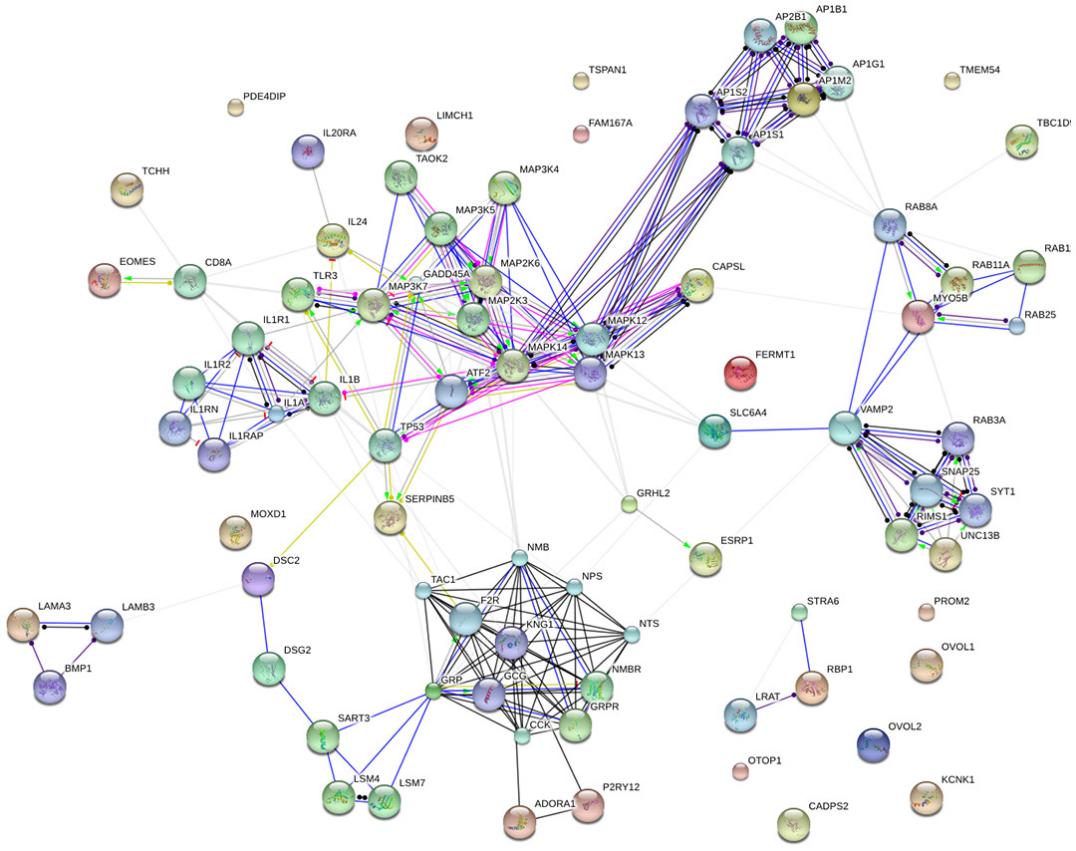
Genetic interaction network associated with breast cancer liver metastases basing on String platform In this figure, each circle represents a gene (node) and each connection represents a direct or indirect connection (edge). Line color indicates the type of interaction evidence and line thickness indicates the strength of data support.

A genetic interaction network was constructed from the 48 differentially expressed genes using the String platform future analysis (Figure 2). The interaction network consisted of 84 nodes and 237 edges. The average node degree was 5.64. The network was reliably robust with a clustering coefficient (cc) of 0.598 and protein-protein interaction (PPI) enrichment *p* value of zero.

### GO analysis of the differently expressed genes

Molecular function analysis by the GO con-sortium database revealed that most of the differently expressed genes regulated protein binding and kinase activity (Table 2). Besides, the major biological processes associated with the liver metastases were positive regulation of cell communication, MAPK cascade, signaling, and protein kinase activity (Table 3).

**Table 2:** Molecular function analysis of the genetic interaction network associated with liver-aggressive explant in terms of Gene Ontology (GO)

GO ID	Molecular Function	Observed Gene Count	*FDR*
GO.0004702	receptor signaling protein serine/threonine kinase activity	15	3.13E-21
GO.0005515	protein binding	7	2.03E-05
GO.0004708	MAP kinase kinase activity	41	2.41E-05
GO.0017137	Rab GTPase binding	5	2.74E-05
GO.0031489	myosin V binding	6	0.000307
GO.0017022	myosin binding	4	0.000381
GO.0004709	MAP kinase kinase kinase activity	5	0.000518
GO.0005488	binding	4	0.00169
GO.0017075	syntaxin-1 binding	59	0.00354
GO.0004707	MAP kinase activity	3	0.00402
GO.0004674	protein serine/threonine kinase activity	3	0.00636
GO.0004946	bombesin receptor activity	9	0.0113
GO.0005102	receptor binding	2	0.0128
GO.0004908	interleukin-1 receptor activity	14	0.018
GO.0019905	syntaxin binding	2	0.0215
GO.0019899	enzyme binding	4	0.0253
GO.0004871	signal transducer activity	15	0.032
GO.0005179	hormone activity	16	0.0377
GO.0060089	molecular transducer activity	4	0.0377
GO.0086083	cell adhesive protein binding involved in bundle of Hiscell-Purkinje myocyte communication	17	0.0377

Abbreviations: FDR, false discovery rate; GO, Gene Ontology.

**Table 3:** Biological process analysis of the genetic interaction network associated with liver-aggressive explant in terms of Gene Ontology (GO)

GO ID	Biological Process	Observed Gene Count	*FDR*
GO.0051046	regulation of secretion	21	5.45E-10
GO.0080134	regulation of response to stress	28	6.97E-10
GO.1903530	regulation of secretion by cell	19	4.53E-09
GO.0051047	positive regulation of secretion	15	8.72E-09
GO.0032101	regulation of response to external stimulus	20	1.24E-07
GO.0032879	regulation of localization	31	1.24E-07
GO.0051049	regulation of transport	27	1.24E-07
GO.0051050	positive regulation of transport	20	1.24E-07
GO.0031347	regulation of defense response	18	3.95E-07
GO.0010647	positive regulation of cell communication	25	4.18E-07
GO.0060341	regulation of cellular localization	22	4.18E-07
GO.0043410	positive regulation of MAPK cascade	14	8.81E-07
GO.0014047	glutamate secretion	6	1.17E-06
GO.0050690	regulation of defense response to virus by virus	6	1.38E-06
GO.0023056	positive regulation of signaling	23	1.79E-06
GO.0051650	establishment of vesicle localization	10	2.00E-06
GO.0046717	acid secretion	7	3.36E-06
GO.0001934	positive regulation of protein phosphorylation	17	5.02E-06
GO.0016079	synaptic vesicle exocytosis	37	3.10E-13
GO.0045860	positive regulation of protein kinase activity	11	3.55E-13

Abbreviations: FDR, false discovery rate; GO, Gene Ontology; MAPK: mitogen-actived protein kinase.

### Signaling pathways involved in breast cancer liver metastasis

Table 4 shows the signaling pathways involved in breast cancer liver metastases by the KEGG database. The major signaling pathways included the MAPK, NF-kappa B and VEGF signaling pathways that maybe critical for the distinct pathological stages of liver metastasis.

**Table 4:** Signaling pathway analysis of the genetic interaction network associated with liver-aggressive explant in terms of Gene Ontology (GO)

Pathway ID	Signaling pathway	Observed Gene Count	*FDR*
4010	MAPK signaling pathway	16	1.42E-12
4668	TNF signaling pathway	9	7.29E-08
5014	Amyotrophic lateral sclerosis (ALS)	7	1.26E-07
4750	Inflammatory mediator regulation of TRP channels	8	3.45E-07
4380	Osteoclast differentiation	8	1.45E-06
5140	Leishmaniasis	6	1.24E-05
4721	Synaptic vesicle cycle	5	0.000104
4664	Fc epsilon RI signaling pathway	5	0.000156
4660	T cell receptor signaling pathway	5	0.000787
5146	Amoebiasis	5	0.000993
4060	Cytokine-cytokine receptor interaction	7	0.00133
4722	Neurotrophin signaling pathway	5	0.00145
5160	Hepatitis C	5	0.00206
4015	Rap1 signaling pathway	6	0.00207
4911	Insulin secretion	4	0.00355
4728	Dopaminergic synapse	4	0.0148
5131	Shigellosis	3	0.0148
4370	VEGF signaling pathway	3	0.0155
5162	Measles	4	0.0162
5120	Epithelial cell signaling in Helicobacter pylori infection	3	0.0194
5222	Small cell lung cancer	3	0.0351
4064	NF-kappa B signaling pathway	3	0.0384
5168	Herpes simplex infection	4	0.0384
4723	Retrograde endocannabinoid signaling	3	0.0473

Abbreviations: FDR, false discovery rate; GO, Gene Ontology.

## DISCUSSION

Breast cancer liver metastasis is a complex process that includes tumor cell dissemination from the primary tumor, transit through the blood or lymphatic system, and proliferation in liver. Underlying this complex multistep process is a sophisticated network of molecular events. In this study, we generated, for the first time, a comprehensive genetic interaction network from the microarray gene expression profile to identify the molecular mechanisms involved in breast cancer liver metastases. The results suggested that MAPK, NF-kappa B and VEGF signaling pathways are significantly associated with distinct stages of breast cancer liver metastasis.

Dissemination of carcinoma cells is the initial step of the metastasis, which is initiated by epithelial-mesenchymal transition (EMT) program during which tumor cells acquire mesenchymal features and lose epithelial properties [8, 9]. The complex molecular events during EMT are initiated and controlled by signaling pathways that respond to extracellular cues. The transforming growth factor-β (TGF-β) signaling family plays a predominant role in EMT [10]. Moreover, the MAPK signaling pathway is required for the initiation of TGF-β induced EMT [11, 12]. In addition to TGF-β family proteins, tyrosine kinase receptors (RTKs) play a key role in the trans-differentiation process, further highlighting the importance of MAPK signaling [13]. MAPK pathway inhibitors have been used clinically for many cancers, including breast cancer [14]. In addition, NFκB is an important regulator of the expression of various proteins involved in the immune response [15].

After successfully disassociating from the primary tumor, metastatic carcinoma cells traverse the blood or lymphatic system, during which they interact with several cell types including platelets, neutrophils, monocytes, macrophages, and endothelial cells [16]. The circulating tumor cells also interact with platelets [17] and high platelet counts are associated with poor prognosis in carcinomas [18]. Recent studies have revealed that platelets alter the fate of circulating cancer cells [19]. Platelet-tumor cell contacts and platelet-derived TGF-β synergistically activate the TGF-β/Smad and NFκB pathways in cancer cells enabling their transition to an invasive mesenchymal-like phenotype, thereby enhancing metastasis [20]. Inhibition of NFκB signaling in cancer cells or ablation of TGF-β1 expression in platelets protects against lung metastasis *in vivo* [20].

In the liver, a pre-metastatic niche is established by VEGFR^+^ bone marrow progenitors before the arrival of tumor cells [21]. In fact, the initial events during the development of metastasis are VEGF-dependent [22]. Once the metastatic cancer cells survive in the new environment, they undergo colonization before the onset of the final process of malignancy. In general, a tumor requires angiogenesis to grow beyond 1-2 mm in size. In the initial pre-vascular phase, the size of the tumor does not exceed a few millimeters, but, neo-vascularization results in rapid growth of the tumor. Vascular endothelial growth factor (VEGF) is a key regulator of angiogenesis, which stimulates endothelial proliferation and migration, inhibits endothelial apoptosis, and increases vascular permeability and vasodilatation [23]. VEGF-targeting therapy has shown significant benefits in the treatment of metastatic breast cancer [24, 25]. In conclusion, based on the genetic interaction network, we identified MAPK, NF-kappa B and VEGF signaling pathways as key regulators of breast cancer liver metastasis.

## MATERIALS AND METHODS

### Microarray dataset resources

Microarray dataset with the accession number GSE62598 was downloaded from Gene Expression Omnibus (GEO). In this study, the authors examined if the propensity of breast cancer cells to metastasize to liver was associated with distinct patterns of immune cell infiltration [26]. Total RNA was extracted from 4T1 parental and individual metastatic sub-populations. The mRNA array was performed on Agilent-014868 Whole Mouse Genome Microarray 4×44k G4122F platform.

### Analysis of differentially expressed genes

The gene expression profiles of metastatic tumor cells versus disseminated tumor cells were normalized by log_10_ transformation after normalization. Then, Significance Analysis of Microarrays software (SAM, http://statweb.stanford.edu/~tibs/SAM/) was used to produce a cluster of up- or down-regulated genes [27].

### Genetic interaction network construction

Genetic interaction network was constructed using the String consortium database (http://string-db.org/). In addition, to identify the pathways involved Gene Ontology consortium (GO, http://www.geneontology.org/) and Kyoto Encyclopedia of Genes and Genomes (KEGG, http://www.genome.jp/kegg/) functional enrichment analysis was performed using the Database for Annotation, Visualization and Integrated Discovery (DAVID, https://david.ncifcrf.gov/).

### Statistical analysis

According to a previous publication [28], gene expression was considered significant if the threshold of false discovery rate (FDR) ≤ 5% and fold change ≥ 2. For GO and KEGG enrichment analysis, biological process, molecular function and signaling pathways, *p* ≤ 5% was considered significant.
